# How to observe business operations: An empirical study of family business

**DOI:** 10.1371/journal.pone.0267223

**Published:** 2022-04-21

**Authors:** Tsu-Cheng Chou, Hsi-Peng Lu

**Affiliations:** 1 Graduate Institute of Management, National Taiwan University of Science and Technology, Taipei, Taiwan, ROC; 2 Department of Information Management, National Taiwan University of Science and Technology, Taipei, Taiwan, ROC; Hacettepe Universitesi, TURKEY

## Abstract

The direct observation method is commonly used for data collection in family business case studies. Nevertheless, in research on family business succession that is mainly based on retrospective data, it is difficult to directly observe an event or situation at a given time. This paper thus first explores the application of observation data in the published research results of some family business case studies through a literature review. It then describes our observation methodology, i.e., sampling process, method, observation process, and reevaluation of our interview data, through a case study. Finally, the conclusion offers suggestions for using these observation methods, i.e., employing different types of observation, by considering running time and financial cost, familiarizing observers with various observation occasions, and seeking the assistance of relevant professionals for a research topic.

## Introduction

The case study is the most commonly used qualitative research method in family business research [1]. There are six types of sources of evidence for case studies: documents, archival records, interviews, direct observations, participant observations, and physical artifacts. No single source has comprehensive advantages, and different sources are complementary [2]. Interviews are the most commonly used data source for family business case studies [3, 4]. They are considered the most appropriate method when researchers are trying to understand what respondents consider essential or when research topics focus on deep-rooted values or beliefs and the complex dynamics of family and business [1]. However, family business owners and managers are often reluctant to provide sensitive information about their company and family and may be less willing to participate openly in research projects without higher levels of researcher engagement [5]. Narrators may gloss over stories, provide an embellished account of what happened or exaggerate the events and facts of intended and realized strategies [6]. It is thus difficult to prevent memory distortion [7] or inaccuracy [4], as well as any deviation caused by social image and self-esteem [3].

Family businesses are often privately owned, which restricts the type of secondary data access that is readily available for publicly listed corporations [5]. rthermore, files and records are often highly variable in quality, with great detail in some cases and virtually none for other programmatic components [8]. One way to reduce data inaccuracy is to calibrate the data sources of multiple respondents. Another essential method is to combine interviews with direct observation [1] to form an evidence database for research.

Family business succession has been one of the hot topics in family business research since the 1950s [9–11]. Hence, Le Breton-Miller et al. [10] have proposed an integrated, four-stage model for the succession process in a family business: defining the ground rules, training potential successors, selecting the successor, and passing the baton at the successor’s establishment. Because succession is a process involving numerous activities over a protracted period [12], its temporal factors are an inherently important consideration [13]. The critical approach to evaluating narratives in family business entails a rather traditional qualitative analysis, where scholars inductively analyze and interpret a narrative to extend, understand, or develop theoretical frameworks [14].

Family business succession is a long-term process involving three elements: the predecessor, the successor, and the succession process [12, 15]. Undoubtedly, the source of evidence for its analysis should mainly be the retrospective interview data of crucial persons [3, 4, 16]. Accordingly, since direct observation is a record of real-time phenomena, conducting it while studying family business succession amid the mutual calibration of data accuracy is this paper’s focus. Based on our direct observation during our research on the social capital succession of family enterprises in the Taiwanese machinery industry, this paper discusses the following: (1) the application of the direct observation method in a case study and (2) how to calibrate observation data and interview data.

The research period was from May 2017 to August 2020. The case study method was used to observe the operation of 8 family businesses, interview 12 incumbents and successors, and examine the relevant documents of these family businesses. Although we obtained some exemplary papers on observational execution methods and analytical methods, there was a shortage of papers that met the transparency criteria for qualitative research. Therefore, this article offers some suggestions on the application of observational analysis in the future, including the research design of direct observation, the implementation steps, and the use of mixed-research methods, which are expected to help researchers improve the transparency and rigor of their case studies in their future research and publications. Therefore, this article first describes the transparency criteria for qualitative research and the implementation of direct observation methods. Next, it reviews some early and recent salient journal papers and then explains the process of our empirical research. Finally, it presents our findings, conclusions, and recommendations.

## Literature review

### Quality of observation research

Regarding the desirability of replicability, this is a potentially contentious issue in qualitative research, in contrast to quantitative research [17]. Reay [18] offers several suggestions for publishing high-quality qualitative research. Her recommendations include (1) ensuring access to sufficient, high-quality data, (2) composing an appropriate research question to guide the article, (3) grounding the study in the relevant literature, (4) explaining the methods in detail, (5) telling an intriguing empirical story, (6) articulating a convincing theoretical story, and (7) providing a clear contribution to the family business literature.

For case study methodology, Maxwell [19] identifies the following four measures to improve a study’s validity: intensive and long-term involvement; creation of rich data, i.e., complete transcription of interviews; triangulation, involving various informants and methods; and a multiple-case design to allow comparison across cases.

Concerning the practical application of research procedures, Aguinis and Solarino [17] offer best-practice recommendations—approximately 12 transparency criteria that authors can use when conducting their work. These requirements include research design (i.e., qualitative method, research setting, position of researcher on the insider–outsider continuum, sampling procedures, relative importance of participants/cases), measurement (i.e., documenting interactions with participants; saturation point; unexpected opportunities, challenges, and other events; management of power imbalance), data analysis (i.e., data coding and first-order codes, data analysis and second-and higher-order codes), and data disclosure (i.e., raw material availability). They emphasize that transparency is a continuous variable and a matter of degree. Therefore, the more fully met criteria there are, the better [17].

### Observation study execution procedure

When researchers visit their case study site, opportunities for direct observation are created [2]. Direct observation requires them to spend time observing and experiencing the operations of a company or organization, and these observations can be time-consuming and expensive. This kind of data source is particularly suitable for studying all aspects of organizational culture. Only by experiencing corporate activities can the fundamental values and ideas commonly held by the members of an organization be understood [1].

Direct observations of phenomena, behaviors, or objects’ occurrence processes that lack an oral report by interviewees can prevent the influence of interviewees’ screening of information or incomplete expression. Thus, the data obtained are highly reliable, and the distortion rate of data conversion is minimized [5]. Furthermore, direct observation can record actual real-time events and the contexts of their occurrence [2], which is its most significant advantage.

There are few research objects suitable for direct observation in family business research. Observations must be made of specific behaviors or objects [8, 20]. Typically, however, only performed or explicit behaviors can be observed, but one cannot understand the internal thinking process of the relevant research subjects. Therefore, to address a research question, the observer may have to infer and interpret the observation results, and such interpretations may be entirely subjective [21]. In addition, researchers must witness the occurrence process of an event as it happens. Predicting the timing of events makes it difficult to observe them. The research subjects and events that can be observed simultaneously are limited, making the time cost of observation very high [2, 8].

Therefore, the observation method is more suitable for studying nonhistorical situations. As Yin [2] has pointed out, assuming that the phenomenon to be studied is not entirely historical, some information about appropriate behaviors and environmental conditions can be obtained through observations. Such observations can then be used as another data source in a case study.

For reliability, Yin [2] suggests creating a case study protocol that details the exact procedure that the cases have been approached with. Such a protocol should include field procedures, e.g., case sites, credentials, and schedules. Observation studies are conducted in steps to achieve the regularity and accuracy required for direct observation [20, 22]. These steps include (1) selecting a site based on its reflection of a theoretical issue, not because it more or less represents an issue of immediate concern or is simply convenient; (2) gaining entrée—when the subject of observation is unfriendly, the gatekeepers should be contacted to obtain their approval and support; (3) starting the observation and spending some time becoming familiar with the local environment and how to interact within it; (4) using the recording method that can best help the researcher to retrieve and analyze the collected data; (5) finding some patterns in the observation for further observation or extended study; and (6) continuing to observe until theoretical saturation is reached.

Moreover, researchers’ actual strategies for observation include entering the observation scene or contacting individuals through network referrals, concisely stating the purpose of the study and the intent of the observations, having sufficient knowledge of the research field and subject, showing the ability to conduct research, and graciously asking for cooperation and negotiating a convenient time and place for observation [2, 20, 21].

### Literature on observation studies

A well-rounded study should clearly state its observation procedure to confirm the reliability and validity of its case study. Aguinis and Solarino [17] have searched and analyzed 52 articles involving “interviews with elite informants” that were published in *Strategy Management Journal* from 2000 to 2017. Overall, and across the 12 criteria, none of the 52 studies are sufficiently transparent to facilitate exact replication. Furthermore, few studies list all the sources they used, while only one-third of the total sample identify the nature of their sources.

Leppäaho et al. [23] have provided similar research findings. They conducted an in-depth qualitative content analysis of 75 qualitative family business case studies, published between 2000 and 2014 in high-quality academic journals. Overall, these applications of the case study method appeared to lack transparency.

Accordingly, in the present study, we first reviewed 26 qualitative research papers on family businesses by Fletcher et al. [24]. Among them, ten used direct observation for collecting data, and only two provided specific explanations on their practical application of direct observation. For example, Kotlar and De Massis [25] investigate goal setting among 19 family businesses, having obtained their research evidence through interviews, observations, and documents, and employ a table to detail the participation of different research subjects in family and business meetings (board meetings, family meetings, and unscheduled meetings). In total, 114 meetings were observed across 19 companies, the least of which was three meetings and the most 12.

Additionally, Nordqvist and Melin [26] investigate strategic planning practices in three medium-sized and multigenerational family firms and explain how to observe family businesses as follows:

*The empirical material of the overall project involves 98 face-to-face interviews (with key actors*, *such as owners*, *managers*, *consultants*, *board members*, *family members*, *accountants*, *former managers)*, *observations of 10 meetings where strategic issues were treated (e*.*g*., *board meetings*, *top management team meetings*, *strategic planning meetings*, *strategy away days)*, *casual conversations*, *site visits and secondary sources such as company reports*, *minutes from board and top management team meetings and strategic plans*. *All interviews were audiotaped and transcribed verbatim*. *Detailed notes were taken during all observed meetings*

[26, p. 18].

The internal meetings of enterprises are the most observed occasions in case studies of enterprises. However, most studies provide only a brief description of the implementation process of these practical sessions, with limited descriptions of the meeting occasions, frequencies, observation focus, and collected data:

*The fieldwork comprised 95 interviews*, *observations of several meetings*, *and many site visits to the firms where we interacted informally with family and firm-related individuals*

[27, pp. 55–56].

*We conducted these two case studies over two years*, *with more than ten interviews in each firm*, *supplemented with several informal talks with key actors and participation in several meetings*. *Thus*, *each study covers a period of more than ten years*, *where we both reconstructed the near history and observed the ongoing change processes in real time*. *Together with documents and notes from observations and informal talks*, *the transcribed interviews represent the basis for our interpretations of the family business culture and its impact on entrepreneurial processes*

[28, p. 198].

Case observation is often carried out simultaneously with interviews. For example, a case study on the social network of eight Vietnamese nail salons in London mentions that its authors had observed the cases during their discussions without specifying the frequency and manner of the observed cases:

*The interviews with the owner-managers took place in the nail shops*, *enabling observations of the business to be made*. *By interviewing and observing these different aspects of the supply chain*, *it was possible to cross-check and triangulate data from various sources and build up a picture of how the sector operated*

[29, p. 384].

Several studies only mention case observations and do not explain how to conduct observations; that is, they lack any discussion of observation subject, location, number, or other research processes:

*his research arises from a self-enriching process of reading*, *analysis*, *observation*, *interviewing*, *and writing*

[30, p. 268]

*Data were collected over two years (2003 and 2004) from a range of sources*, *including interviews of senior managers from each firm (34 in total)*, *observations*, *notes from 22 field visits*, *questionnaires*, *and firm documents*

[31, p. 155].

Second, we examined whether the application of observational methods in the family business research literature of the last five years has improved compared to earlier periods. We found several studies that once again only mention case observations without explaining how they conducted their observations:

*We took field notes*, *documenting our company visits and observations*. *The research output of this stage is not presented here but was extremely useful for the research team to contextualize the participants’ narratives of the succession process collected in the interviews*

[32, p. 583].

*The authors started by independently examining the data in interviews*, *observation notes*, *and documents*. *Then*, *a coding process was carried out by reading and rereading transcripts*, *notes*, *and documents and then using codes for sentences or paragraphs in order to organize data*

[33, p. 173].

We also found that observational methods are often used as an incidental data source and that some papers describe the interview process in far more detail than the observational process entails:

*We integrated data collected from a wide range of sources*: *interviews*, *historical records*, *financial data*, *sustainability data*, *and direct observations to triangulate (to adopt different angles in observing the same phenomenon)*. *We chose to use the semistructured interview because of its high degree of flexibility and because it offers the opportunity to address themes that can come to light during the semistructured interviews*. *Data collection was conducted from March 2015 to April 2015*. *The interview transcriptions*, *field notes*, *and documentary analysis were coded into key themes*

[34, p. 977].

Some studies briefly account for the frequency of observations but do not explain how they conducted their observations, omitting their observation subject, location, number, or other research processes:

*Our single-case study framework allowed us to conduct participant observations*. *During this period*, *one researcher systematically attended all meetings organized by the consulting firm with the different stakeholders (approximately one day every two months)*

[11, p. 260].

*Data processing was performed using a handwritten copy of our observations*, *interviews*, *and secondary data sources*

[11, p. 261].

Third, we also obtained published articles with high transparency that could serve as examples for future researchers. For instance, Wielsma and Brubbinge [35] conducted a longitudinal single case study. They detail their procedures for data collection as follows:

*The data were collected between September 2015 and September 2017*. *During this time*, *the company was visited in intervals ranging from one week to four months*.

*Interviews were recorded and transcribed*, *while note-taking was used for the informal talks*. *The participants in the project included family members (six individuals from three generations) and nonfamily employees (twelve individuals)*. *Additional data were obtained through nonparticipant observation of staff meetings*, *review of company documents (including the website)*, *and review of publications from third parties*. *The use of multiple sources during data collection enabled the triangulation of information*

[35, p. 41].

Especially the way the authors use the table [35, p. 42], which lists the data collection methods, periods, data sources, and durations, including nonparticipatory observations and other research methods. The research procedures and data are thus highly transparent and reproducible.

The following problems are found in the above research that has implemented the observation method to collect family business case data. First, the collection and design of observation data are accompanied mainly by interviews, and only a few works actively plan the occasions, frequencies, and focus of observations. When determining an observation site, although directly related to a research topic, most observations are based on an interview location. Second, the most frequent observation context is the internal meeting of a company. However, the interaction between a company and the outside world cannot be observed within the company. Third, only some of the observations clearly state the frequency and nature of the observation meetings, and most of them lack an explanation. Fourth, the observation recording method does not explain the use of an open structure or a closed record table. Fifth, it is impossible to grasp how most studies’ observation data are combined with other interviews and documents to form the evidence chain of their information. Finally, we have not discerned any discussion of how to overcome difficulties and obtain the support of an observation object. Accordingly, family business case studies seem to be dominated by interview data, while observation data are only incidental.

For a researcher interested in the direct observation of family business cases, it is unfortunate that he or she cannot refer to the existing empirical research literature to learn the relevant application steps. These include establishing the precautions of the observer, selecting a site, obtaining the support of the subject of observation, and recording the observation(s).

## Materials and methods

### Case study

The methodology for this project was the case study approach. A case study is an appropriate data collection method if the goal is to understand how or why a phenomenon occurs [23, 36]. In the context of family firms, case studies are helpful because data come from various sources, allowing scholars to capture multiple perspectives [1]. In addition, this approach enables a deeper and more meaningful understanding of real-life events in a business context [2].

To increase our understanding of succession in family business, a qualitative multiple-case study methodology was identified as the most appropriate means to answer our how and why questions [1]. The research period was from May 2017 to August 2020. The case study method was used to observe the operation of 8 family businesses, where we interviewed 12 predecessors and successors.

### Theoretical sampling

In this study, we selected cases with theoretical sampling by choosing the predecessors and successors of representative family businesses. All the chosen family businesses were directly contacted by the researcher. Hence, the researcher was able to determine whether the cases would meet the research purposes by gauging the family businesses’ internal functions and how their two relevant generations interact.

Purpose sampling should be conducted in qualitative research because how much data are collected from how many subjects are irrelevant [37]. Instead, it is more important to collect correct information that is sufficiently rich to enable a better understanding of the case phenomenon’s meaning [4]. This study therefore entailed the following preselection criteria: first, the cases’ capital or business volume should be relatively large and unique in their market to show a value of succession. The interviewed managers should be responsible for all or most of their company’s operations to affirm the significance of the research. Therefore, the sampling criteria for this study included two levels: organizational and individual.

The conditions regarding organization level were mechanical industry, small-and-medium enterprise (SMEs), and family business. In Taiwan, the robotic industry is defined by cases that involve members of the Taiwan Association of Machinery Industry (TAMI). According to the Taiwanese government, SMEs must contribute capital below NT $100 million (US $3.57 million) or have fewer than 200 employees. Finally, a family business is an enterprise where family members obtain ownership and are responsible for operations.

At the individual level, we believed it was worthwhile to interview the predecessors and successors in the same enterprises to conduct data cross-validation. In addition, the successors needed to have more than simply a title or position; they needed to be among the top management team that is responsible for all or most operations in their company.

There is no accepted ideal number of cases. Eisenhardt [38] suggests that it is challenging to generate theory from fewer than four cases, but more than ten make it challenging to manage and analyze data. The appropriate number of cases will therefore be determined by how each case incrementally contributes data [39].

The machinery industry is one of the three most significant industries in Taiwan. Most companies in this industry are family-owned SMEs. Before formal invitations were sent, the researcher asked the chairman of TAMI to solicit support for this study. This allowed the researcher to select the case study companies with purpose sampling.

Hence, we selected eight family businesses with successful succession and spoke with 12 interviewees. All eight companies are market leaders or uniquely positioned in the marketplace. Passing the torch to the next generation is worthwhile for these companies. The capitalization of these companies ranges from US $357,000 to US $6.6 million. Their number of employees ranges from 30 to 200 people. The youngest company is 18 years old, and its founder is passing the business to the second generation. The oldest company has an operating history of 70 years, and the third generation has already inherited it from the second generation. All the participants met the research criteria for a multiple-case study. Table 1 summarizes the background of these eight companies, which are sizable, in the same sector, and align with the standards suggested by Miller & Le Breton-Miller [40] in their review of research on family businesses.

**Table 1:** Case backgrounds.

Case	Characteristics of the company	Time of foundation	Capital	Number of employees	Import and export sales
A	Rubber machines	1974	2.0 million	80	40% import sales
	Top 5 in Taiwan				60% export sales
B	Molding machines	1953	6.6 million	200	10% import sales
	Top 1 in Taiwan				90% export sales
C	Machinery brakes	1948	1.28 million	40	30% import sales
	Top 1 in Taiwan				70% export sales
D	Old brand of CNC lathes	1958	4.28 million	100	5% import sales
					95% export sales
E	CNC lathes	1988	6.42 million	140	50% import sales
	Top 5 in Taiwan				50% export sales
F	Handling equipment	1955	357,000	30	35% import sales
	Top 1 in Taiwan				65% export sales
G	Robotic arms	1990	892,860	54	40% import sales
	Top 1 in Taiwan				60% export sales
H	Tool magazine	1980	964,300	160	30% import sales
	Top 1 in Taiwan				70% export sales

Capital units have been converted into US dollars.

### Data collection

This study involves three data sources: interviews, direct observations, and documents. Family business succession is a long-term process [12], and the interaction between predecessors and successors may start during family life instruction before the successor matures. These complex, long-term interactions have many details that are unknown to outsiders. Information can be obtained through retrospective interviews. In addition, researchers often have close contact with the interviewed companies and interviewees in their work, offering researchers many direct observation opportunities to collect information beyond interviews. These include observing the interaction of family members in a case enterprise, evaluating the division of labor among family members and the interaction among peers at the exhibition site, and monitoring how a predecessor leads the next generation to expand horizontal and vertical social relationship capital during fellowship activities. This study also uses document data from the *Economic Daily News* (Taiwan) database, books, and magazines.

**Fig 1:**
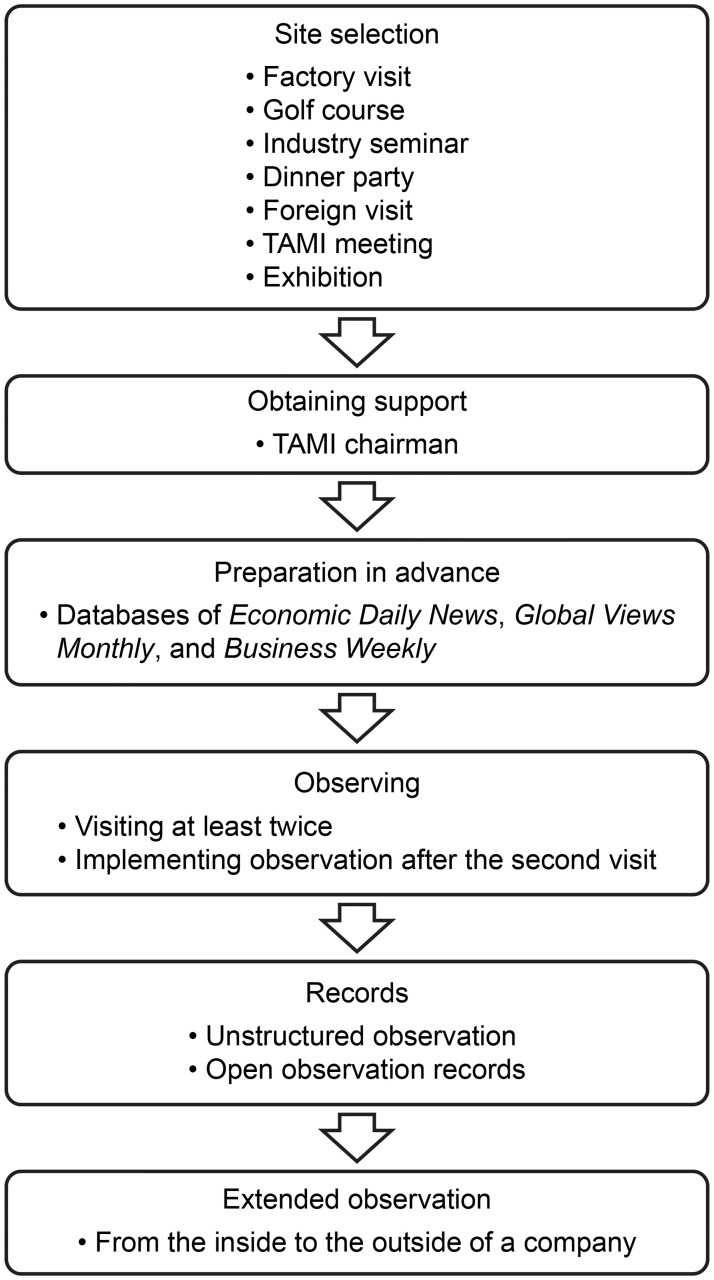
Observation steps.

This article mainly discusses the use of observational methods, and thus the steps we used to conduct our observational data collection are described below and illustrated in Fig 1.

#### Site selection

In this study, interviews for seven of the eight case enterprises were conducted in their factory to facilitate the observation of factory operations. Nevertheless, the observation field was not limited to the inside of each case company. We also needed to collect the external social capital succession data of the subjects according to the research framework. Therefore, we also conducted observations at golf clubs, a meeting of TAMI’s board of directors and supervisors, industry dinner parties, domestic and foreign exhibition sites, industry symposiums, and other forms of external enterprise cooperation.

#### Obtaining support

Researchers must attain a high level of trust when studying family businesses to potentially improve response rates and accuracy when eliciting information through direct means, such as surveys [35, 41]. The critical factors for the successful interviews and observations in this study were that the researcher had preexisting friendships within the machinery industry and the support of the chairman of TAMI.

#### Preparation in advance

We searched the relevant reports of the case businesses through the databases of *Economic Daily News*, *Global Views Monthly*, and *Business Weekly*. In addition, we learned the backgrounds of the cases and interviewees to enrich our preparation work before the interviews.

#### Starting observation

All cases were contacted directly by the researcher via telephone, who called to explain the purpose of the study and confirmed whether they agreed before making formal visits. During the first visits, the researcher indicated the research relationship with the interviewees, the research purpose, and the research outline. Research interviews and observations were conducted after the second visits. The intervening period from the first formal visit to the enterprise observation was often more than three weeks. The research period was from May 2017 to August 2020.

#### Observation records

In this study, unstructured observations and open observation records were used to plan the field or direction to be observed, but the detailed items were not established in advance. After determining whether the observed information was helpful for the study, the key points were recorded in a notebook.

#### Extended observation

Once we discerned some patterns in our observations, we asked whether there were other suitable places for extending our observations through activities, itinerary visits, industry seminars, golf parties, or other events. There were thus a total of eight factory visits, three golf parties, two industry seminars, three exhibitions, one foreign visit, one dinner party, and one board of directors and supervisors meeting. Hence, there were seven different observation types, 19 observation occasions, and 26 observation records (see S1 Appendix).

### Observation guide

The observation guide is a conceptual framework that we developed via the research literature and research objectives. It includes (1) the size of the case enterprise, including the production equipment and products that are used to confirm the enterprise’s research value; (2) the roles played by the predecessor and successor in the current stage of family business succession; (3) the succession of the internal social capital of a family business (i.e., how to build a human resources team, internal trust relationship, shared vision, and standard language); and (4) the succession of the external social capital of a family business, i.e., vertical social capital, horizontal social capital, and social relationship capital, such as its relationships with customers and suppliers, interaction with peers, inheritance of industrial contacts, and relationships with industry associations, governments, and media.

### Research ethics

Observational research pays special attention to ethical issues because it involves entering the private realm of the case study. Therefore, the following guidelines were strictly followed during the observation period [8, 22]: (1) showing identity—at any site, the researcher stated that the goal of the research is the composition of a doctoral thesis and clearly defined the researcher’s role. Thus, the identity and purpose of the study were known to any case under observation, both in the public and private spheres. (2) Informed consent—with the permission of the case enterprise operator, the researcher entered the enterprise for observation. In the first meeting, the purpose and implementation of the study were clearly stated, and permission was obtained. After the second visit, the researcher began to conduct observations. (3) Commitment to privacy and confidentiality—all the case enterprises and interviewees in the study were treated anonymously. All data will be used for research purposes only and will not be disclosed. (4) Equal and mutually beneficial research relationships—observational research requires researchers and participants to establish a deep bond that is connected, shared, and equal. The cases thus provided a wealth of information to facilitate discussion in this study. In addition, after completing the study, the researcher promised to provide a reference for the respondents.

## Results: Combining observations with other data

Following Neergard and Leitch [42], the authors started by independently examining the data from interviews, observation notes, and documents. Multiple data sources are a strength of any case study [1, 2]. Data collected from various sources can assist researchers in organizing data sources by establishing a case study database comprising interview results, observation notes, and files to improve the reliability of data and assist researchers with subsequent analysis. Using different data sources, researchers can triangulate data and adopt different angles to observe the same phenomenon [2], thus making their findings more credible and accurate [8, 43]. Accordingly, the present study can be used as an example to illustrate the results of mutual calibration and rechecking via direct observation and other data.

### Succession stage

Case B is the current chairman and the second generation of his family business. He has been in control for more than 30 years. Five years ago, he made his younger brother the general manager and his younger sister the business manager. The observation data show that the interviewee did not receive a single phone call, document, or request during the entire three-hour morning interview in his office. Indeed, the interviewee claimed that he had finally completed the business succession.

Case F stated that his elder brother is the chairman of the board of directors but he is the general manager responsible for the company’s operation. Therefore, he would only ask his brother questions if necessary. According to the observation data, the researcher previously met the chairman of the case enterprise. The chairman stated that his younger brother should be interviewed as a representative, confirming the younger brother’s representativeness of the family business.

### Internal social capital: Family human resources

Case B stated that the second and third generations of the family are the primary sources of human resources, accounting for approximately 10% of the company’s employees. According to the observation data, during a visit to the factory, the interviewee introduced the colleagues on the site who are involved in each production process, including the cousin in charge of factory affairs, the nephew managing business development, and the son-in-law directing the department of research and development, demonstrating that numerous family members work in the enterprise.

Case B stated that he began to reserve human resources for future succession when he was in high school. After his university graduation, he agreed to allow ten classmates to work in his family’s enterprise. They then became essential team members who aided his takeover of the management of the enterprise. According to the observation data, when visiting the factory, the researcher met with two deputy general managers, both of whom were high school classmates of the interviewee and had worked in the company for 40 years, confirming the statement of Case B. The interviews and observations in Case B are the same as those presented in a book [44].

### External social capital: Supplier relationships

Case G (founder) emphasized that outsiders were aware that he paid the most attention to quality, acted according to principles, and never accepted commissions or gifts. The observational data showed that Case G refused to accept the researcher’s gift during the first visit, indicating that Case G’s words and actions were consistent.

Case G stated that the second generation is responsible for business development. According to the observation data, the second generation visited the Taichung Automation Industrial Equipment Exhibition and suppliers and customers, confirming the second generation’s role in the enterprise.

Case C (predecessor) is the second-generation successor, and there are currently eight people in the third generation who are department heads in the enterprise. The third-generation successor led four cousins to visit suppliers and customers at the Plastic Machinery Equipment Exhibition held in Guangzhou according to the observation data. After arriving at the exhibition center, the successor greeted the president and secretary-general of the Machinery Association in the office of the exhibition venue and introduced the work that each person was responsible for in the family business. This therefore confirms the division of labor among Case C’s family members in the enterprise and the firm’s practice of maintaining external social capital with, e.g., suppliers and customers.

### External social capital: Industry social network

Case F stated that his brother serves as the chairman of the board while he serves as the general manager and participates in industrial activities on behalf of the company. In addition, according to the observation data, the younger brother represents the company by serving on TAMI’s board of directors and participating in golf activities.

Case G (predecessor) stated that the two generations of the family business divide the labor. He is responsible for internal production and manufacturing, and his eldest son is liable for business development and the maintenance of external relations. According to the observation data, the son of Case G attended the industry symposium sponsored by the *Economic Daily News* on behalf of the company. The son of Case H also represented his company by participating in the industry symposium held by Taichung City Government. This confirms that the second generation is responsible for maintaining the social relation capital of external social capital.

The machinery industry has a close relationship with people in the financial and economic industry media. When senior news media executives visited the case enterprises, the predecessor typically brought the successors together to receive them and introduce them to the next generation. According to the observation data, this occurred at Cases A, C, G, and H.

Cases D and E are the second-generation successors of enterprises with close relations. They share ideas and resources. According to the observation data, Cases D and E play golf, have dinner, and exchange industrial information almost every week.

## Discussion and conclusions

### Discussion

Based on direct observations of cases of family business succession, this study provides the following discussions regarding the execution method of collecting direct observation data in a case study:

First, different data can be obtained from different observation contexts. Observations of the internal operations of enterprises often accompany interviews. We could therefore confirm whether a company’s operating scale and product attributes met the criteria for sample selection. We could also observe the succession of internal social capital, including the division of a workforce within a company, the trust relationship between members of an organization, a shared vision, and a common language. The external field observations of the case enterprises were mainly conducted to evaluate the following: horizontal social capital of the family businesses, including their interactions with the industry and friendships via the second generation of enterprise successors; vertical social capital, including on-site interactions with suppliers and customers; and social relationship capital, including interactions with government departments, industry unions, and industry media.

Second, there are many business activities; hence, it is difficult to systematically implement case observations. This study was therefore conducted over three years, inside and outside of enterprises in Taiwan, at internal production lines, external exhibitions, golf courses, and dinner parties. The costs of conducting so many case observations are very high, and their implementations are also difficult. However, the data we collected are extremely valuable and cannot be replaced with interview data.

Third, it is necessary to clearly state the recording method for obtaining observation data and how the data will be collated and rechecked with other data sources to form a transparent chain of evidence, which helps strengthen the reliability and validity of the research. Observation is not a random glance. Scientific inquiry entails using observational methods that require disciplined training and rigorous preparation [8]. Such training includes learning how to write descriptively, practicing the disciplined recording of field notes, separating useful details from trivia to obtain the former without being overwhelmed by the latter, and using rigorous methods to validate observations. Moreover, proper preparation has mental, physical, intellectual, and psychological dimensions that must be learned to effectively concentrate during an observation [8].

Accordingly, this article provides five suggestions for applying the observation method in family business case studies, which derive from our findings. (1) Observation design should be included in the research design stage. The researcher should select observation objects, determine the occasion and frequency of observation, design the observation outline, and compose records according to the research question. Observation methods are not incidental and should have a thorough and complete design. (2) Although occasions for observation vary according to research topic, one should pay attention to internal and external observations of the research case. External observation facilitates obtaining multiple pieces of information and can account for any deficiency of inner observation. (3) Long-term observation is necessary. Since corporate activities are very diverse, the time and cost of observations are very high, and it is challenging to implement systematically, it is difficult to obtain sufficient research data in a short period. (4) Researchers should be familiar with various observation environments, have sufficient industry knowledge, and exhibit considerable social skills to harness every occasion to perform an observation. For example, formal industry events, such as association meetings, exhibitions, or forums, and informal events, such as fellowship parties, galas, and dinner parties, are more complex than company meetings. Thus, it is challenging to familiarize oneself with the specific environments of various observation venues. (5) Researchers should "get a ticket" to an observation and attempt to bridge the gaps between academia and industry. Finding professionals with rich connections within an industry can help the observer attain the support and trust of research cases and the opportunity to observe family businesses’ internal and external operations in depth.

Fourth, observations can be both qualitative quantitative. According to the literature and this study, an observation is often accompanied by interviews [2]. If interviews are conducted in respondents’ company or factory, the number of employees in the case company that are observed on site can confirm the company’s size. For example, the brand, quantity, and depreciation of factory production equipment can also be used to evaluate investment funds. The number of container trucks in a factory parking lot can be used to reference shipment turnover. Whether a factory regularly requires overtime can affirm a company’s revenue. Observations at an exhibition site may also have quantitative data. For instance, the size of the booth at an exhibition is often an indicator of a company’s size. The number of visiting buyers can also be used as a reference for revenue. Overall, this approach represents a mixed-method design.

Mixed-methods research utilizes both qualitative and quantitative perspectives in the same study. Here, quantitative survey data were collected for empirical information (existing contextual, relational, and business structure facts), while qualitative data were collected through interviews [45]. Mixed-methods research can therefore add to the knowledge of and generalizability of a case topic. Specifically, researchers can capture the distinct complexities of family business research [46].

Mixed-methods research offers the capability to make the generalizable observations that usually result from quantitative approaches and to combine them with the rich and "thick" descriptions that typically result from qualitative techniques [46]. Moreover, the combination of qualitative and quantitative perspectives can help uncover previously unobserved relationships, generate interesting new insights to advance theory development [47], and help resolve theoretical complexities [46].

Family business researchers face challenges concerning data collection and the availability of specific data sets and are expected to employ greater rigor to ensure uniform applications of methods [5, 46]. Direct observation is an important data source for a case study. However, such observation requires more time and costs than conducting interviews or accessing archives. Hence, its implementation should be more prudent to prevent the failure of research observations to produce useful evidence. On the other hand, if the difficulties of observation implementation can be overcome and observation sites are sufficiently diverse and complete, then data completeness will be fostered and accuracy will be increased, facilitating the construction of a comprehensive research database involving other data sources, which will form a solid evidentiary basis for case studies.

### Conclusions

Qualitative studies are a crucial for researchers to not only answer important research questions but also develop new questions [48]. The case study is a robust methodology that can be employed in an inventive and rigorous way to generate a more fine-grained contextual understanding of family business phenomena and advance research in the field [23].

Interviews are the primary method for conducting case studies. However, family business case researchers could use data other than pure interviews when developing their accounts [23]. Since researchers want to avoid an overreliance on any one type of sample or data, more creative approaches to data gathering and methods are needed. Furthermore, we should encourage future studies on the nonfinancial performance of family firms to use primary data or draw from a combination of different data sources [49].

In this article, we have analyzed how observation studies have been pursued in the family business literature and have offered suggestions on how they may be used in the future to more effectively capture the idiosyncrasies, dynamics, and processes of family businesses. A family business researcher’s predilection is to rely on past literature as a source of inspiration, and thus such sources should be numerous and of varying types [50]. Where researchers find inspiration and clarification can be an essential factor in the success of their theorizing efforts. Observation can and should be a central component when theorizing. Accordingly, we expect family business research to be more analytical and less subjective than most extant studies.

## Supporting information

S1 Appendix Case observation records. (DOCX)
