# Supplementary material for: How to observe business operations: An empirical study of family business
Source: PLoS One. 2022 Apr 21;17(4):e0267223. doi: 10.1371/journal.pone.0267223 (PMC9022868; doi:10.1371/journal.pone.0267223)
Supplement: S1 Appendix — (DOCX) [file pone.0267223.s001.docx]

**S1 Appendix. Case Observation Records.**

| Site | Situation | Meaning coding | Date |
| --- | --- | --- | --- |
| ChinaPlas Exhibition - Guangzhou | The founder and his wife took the second generation to receive buyers at the exhibition site. | External social capital  Vertical social capital | May 2017 |
| Case factory | The founder, his wife, and three daughters had lunch together at the factory every day to discuss the work and the afternoon schedule. | Internal social capital  Internal organization form | June 2017 |
| Case factory | The predecessor introduced a collection of artworks to the news media, and the eldest daughter offered a supplementary explanation. | External social capital  Social relation capital | June 2017 |
| Industry visit - Yangon | The predecessor stated expanding the business in the emerging region would be left to his daughter. | Internal social capital  Internal organization form | September 2017 |
| Case meeting room | The predecessor repeatedly instructed the eldest daughter to express her opinions on the interview topics before adding her ideas. | Internal social capital  Cognitive capital - the common language | April 2019 |
| Case factory | Visiting production facilities. | Case enterprise size | April 2019 |
| Case factory | During the visit, the interviewee's son-in-law (R&D manager), nephew (business manager), and cousin (factory manager) were introduced. | Internal social capital  Internal organization form | May 2019 |
| Case factory | During the visit to the factory, the two deputy general managers of the company were introduced. They were both high school classmates of the interviewee. | Internal social capital  Internal organization form | May 2019 |
| Office of the chairman of the case factory | There were no telephone calls, official documents, or requests for instructions during the interview. However, the interviewee stated that he had handed over the baton completely. | Succession stage - reconstruction period | May 2019 |
| Case factory | The production equipment and products were introduced. | Case enterprise size | May 2019 |
| Case factory | The predecessor led the successors to introduce the factory's products to the researcher and explain the construction process of the new factory. | Case enterprise size  Succession stage - adjustment period | May 2019 |
| ChinaPlas Exhibition - Guangzhou | The successor took four cousins to visit the exhibition and suppliers. | Internal social capital  Internal organization form  External social capital  Vertical social relations | May 2019 |
| Golf course | The researcher met with the second generation of Case D and E at a gala. | External social capital  Horizontal social capital | May 2019 |
| Case factory | The production equipment and products were introduced. | Case enterprise size | May 2019 |
| Meetings of directors and supervisors of TAMI and dinner parties | His peers praised Case D for being more successful than his father. | External social capital  Horizontal social capital | October 2019 |
| Golf course | The researcher met with the second generation of Cases D and E at a gala. | External social capital  Horizontal social capital | May 2019 |
| A year-end dinner party of TAMI | Case E discussed attending the Tainan Machinery Fair. The interviewee stated that he or she had the right to make decisions. | Inheritance stage - reconstruction stage | February 2020 |
| Case factory | The interviewee introduced each production process and stated that he was familiar with each cycle. | 1. Case enterprise size  2. Succession stage - warm-up period | June 2019 |
| Case factory meeting room | The interviewee introduced the chairman (elder brother), and the chairman stated that the interviewee would be interviewed. | Inheritance stage - reconstruction stage | June 2019 |
| Golf course | The interviewee participated in TAMI activities on behalf of the company. | Internal social capital  Horizontal social capital | August 2020 |
| Industry symposium | The successor attended the industry forum organized by *Economic Daily News*. | Internal social capital  Social relation capital | November 2018 |
| Case factory | The predecessor asked the successor to receive the researcher, and the predecessor dominated the conversation. | Internal social capital  Social relation capital | June 2019 |
| Case factory | The production equipment and products were introduced. | Case enterprise size | June 2019 |
| Case factory | The interviewee politely refused the interview gift. | Internal social capital  Vertical social capital | June 2019 |
| Automation industry exhibition - Taichung | The successor represented the company when visiting the industrial equipment exhibition and customers and suppliers. | External social capital  Vertical social capital | July 2020 |
| Industry symposium | The successor attended an industry symposium organized by Taichung City Government. | Internal social capital  Social relation capital | June 2019 |

Note: Because of the spread of COVID-19 after 2020, observation and research were restricted and then stopped.
